# The Effect of the Paleolithic Diet vs. Healthy Diets on Glucose and Insulin Homeostasis: A Systematic Review and Meta-Analysis of Randomized Controlled Trials

**DOI:** 10.3390/jcm9020296

**Published:** 2020-01-21

**Authors:** Małgorzata Jamka, Bartosz Kulczyński, Agata Juruć, Anna Gramza-Michałowska, Caroline S. Stokes, Jarosław Walkowiak

**Affiliations:** 1 Department of Pediatric Gastroenterology and Metabolic Diseases, Poznan University of Medical Sciences, 27/33 Szpitalna Str., 60-572 Poznań, Poland; mjamka@ump.edu.pl; 2 Department of Gastronomy Sciences and Functional Foods, Faculty of Food Science and Nutrition, Poznan University of Life Sciences, 31 Wojska Polskiego Str., 60–624 Poznań, Poland; bartosz.kulczynski@up.poznan.pl (B.K.);; 3 Faculty of Health Sciences, State University of Applied Sciences in Konin, 4 Popiełuszki Str., 62-500 Konin, Poland; agata.juruc@gmail.com; 4 Faculty of Life Sciences, Humboldt Universität zu Berlin, 14195 Berlin, Germany; caroline.stokes@hu-berlin.de; 5 Department of Medicine II, Saarland University Medical Center, Saarland University, Kirrberger Str. 100, 66421 Homburg, Germany

**Keywords:** Paleolithic diet, glucose, insulin, glycated hemoglobin, glucose metabolism disorders

## Abstract

Recently, the Paleolithic diet became popular due to its possible health benefits. Several, albeit not all, studies suggested that the consumption of the Paleolithic diet might improve glucose tolerance, decrease insulin secretion, and increase insulin sensitivity. Therefore, the aim of this meta-analysis was to compare the effect of the Paleolithic diet with other types of diets on glucose and insulin homeostasis in subjects with altered glucose metabolism. Four databases (PubMed, Web of Sciences, Scopus, and the Cochrane Library) were searched to select studies in which the effects of the Paleolithic diet on fasting glucose and insulin levels, glycated hemoglobin (HbA1c), homeostasis model assessment of insulin resistance (HOMA-IR), and area under the curve (AUC 0–120) for glucose and insulin during the oral glucose tolerance test were assessed. In total, four studies with 98 subjects which compared the effect of the Paleolithic diet with other types of diets (the Mediterranean diet, diabetes diet, and a diet recommended by the Dutch Health Council) were included in this meta-analysis. The Paleolithic diet did not differ from other types of diets with regard to its effect on fasting glucose (standardized mean difference (SMD): −0.343, 95% confidence interval (CI): −0.867, 0.181, *p* = 0.200) and insulin (SMD: −0.141; 95% CI: −0.599, 0.318; *p* = 0.548) levels. In addition, there were no differences between the Paleolithic diet and other types of diets in HOMA-IR (SMD: −0.151; 95% CI: −0.610, 0.309; *p* = 0.521), HbA1c (SMD: −0.380; 95% CI: −0.870, 0.110; *p* = 0.129), AUC 0–120 glucose (SMD: −0.558; 95% CI: −1.380, 0.264; *p* = 0.183), and AUC 0–120 insulin (SMD: −0.068; 95% CI: −0.526, 0.390; *p* = 0.772). In conclusion, the Paleolithic diet did not differ from other types of diets commonly perceived as healthy with regard to effects on glucose and insulin homeostasis in subjects with altered glucose metabolism.

## 1. Introduction

Diabetes is a chronic disease and one of the most prevalent public health concerns globally. The current number of people suffering from diabetes exceeds 425 million in the world, and there is still a large number of people who remain undiagnosed [1]. Furthermore, many people do not meet the accepted criteria for diabetes [2], yet their blood glucose test results are too high to be considered as normal and, thus, they are diagnosed as being in the prediabetes stage. These subjects are very likely to go on to develop type 2 diabetes in the coming years if they do not change their eating habits. Diabetes and prediabetes can be screened based on plasma glucose criteria, either with fasting plasma glucose, 2-h glucose concentrations after an oral glucose tolerance test (OGTT), or glycated hemoglobin (HbA1c) criteria [1,2].

The American Diabetes Association’s (ADA) recommendations strongly emphasize that there is no single eating pattern that is best for those with diabetes; however, they do suggest that the diet should mainly be based on products with a low glycemic index and should exclude refined sugars and processed food [2]. Many studies revealed that diets high in protein can improve diabetes-related parameters. For example, after a three-month intervention, Luger et al. [3] observed improvements in losing weight, fasting glucose, and insulin concentrations in a group of subjects consuming 30% energy from protein in comparison with the control group that consumed 15% energy from protein. Results were comparable with a meta-analysis in which the participants with high-protein eating plans had 2-kg greater weight loss and 0.5% greater improvement in HbA1c [4]. The consumption of carbohydrate foods is also a crucial factor in patients with diabetes or in the pre-diabetes stage. Foods containing carbohydrates have a wide range of effects on the glycemic response because they differ in their proportions of sugars, starches, and fiber [5].

The Paleolithic diet, also known as a hunter–gatherer diet or stone-age diet, is an estimated nutritional pattern considered to be typical of people living during the paleolithic era, from approximately 2.5 million to 10,000 years ago. It encourages the consumption of meat, fish, eggs, vegetables, fruits, roots, and nuts, while many cultivated products, such as dairy products, oils, cereals, and legumes (as well as salt and refined sugars) are excluded [6]. A traditional Paleolithic diet contains an estimated 35% of energy from fats, 35% of energy from carbohydrates, and 30% of energy from proteins. Therefore, the Paleolithic diet typically resembles a low-carbohydrate diet. However, the hunter–gatherer diet provides a higher amount of dietary fiber (up to 45–100 g per day) than a low-carbohydrate diet [7]. Recently, the Paleolithic diet received attention due to its possible health benefits [8]. Paleolithic nutrition is suggested to be associated with an improvement in lipid profile and with the reduction of blood pressure [8,9]. Moreover, this type of diet is suggested to have a positive impact on weight loss [10]. Additionally, it has anti-inflammatory benefits and might reduce oxidative stress [11]. It was also suggested that the Paleolithic diet might have a beneficial effect on carbohydrate metabolism and insulin homeostasis [8], thus making it relevant for patients with diabetes. However, the results of studies assessing the effect of the diet on glucose and insulin levels are ambiguous, with conflicting findings being reported [10,12,13,14,15,16,17,18,19,20].

Therefore, the aim of this meta-analysis was to compare the effect of the Paleolithic diet with other types of diets commonly perceived as healthy on glucose and insulin homeostasis in studies conducted in adults with altered glucose metabolism. Because altered glucose metabolism is frequently observed in subjects with metabolic syndrome and metabolic syndrome is also associated with a high risk of progression to type 2 diabetes mellitus [21], this meta-analysis also included studies in which the majority of participants had at least two characteristics of metabolic syndrome.

## 2. Methods

This systematic review and meta-analysis was carried out and reported in accordance with the guidelines of the Preferred Reporting Items for Systematic Reviews and Meta-Analyses (PRISMA, see Supplementary Materials) [22]. Prior to initiating the review process, the protocol was registered with PROSPERO, registration number: CRD42019126412 [23].

### 2.1. Search Strategy

PubMed, Web of Knowledge, Scopus, and the Cochrane Library databases were searched from time of inception until September 2019, using the following MeSH terms: (((“paleo diet” OR “paleolithic diet” OR “stone age diet” OR “caveman diet” OR “hunter–gatherer diet”) AND (“insulin” OR “insulin resistance” OR “hyperinsulinism” OR “glucose metabolism disorders” OR “hypoglycemia” OR “hyperglycemia” OR “blood glucose” OR “diabetes mellitus” OR “glucose intolerance” OR “glucose tolerance test” OR “glycated hemoglobin A” OR “prediabetic state”)) NOT “animals”). In addition, hand searches of the reference lists of included papers identified further potential studies not captured in the electronic database searches. No language restrictions were applied.

### 2.2. Study Selection

Original studies were included if they met the following inclusion criteria:

- Types of studies: randomized controlled trial (RCTs; parallel or crossover), irrespective of publication status;
- Types of interventions: Paleolithic diet (regardless of the duration of the intervention) versus another type of diet (e.g., the Mediterranean diet, diabetes diets, national dietary recommendation);
- Population: studies conducted in humans with glucose metabolism disorders (diabetes mellitus (criteria for the diagnosis: fasting plasma glucose concentrations ≥126 mg/dL (7.0 mmol/L) or 2-h glucose levels ≥200 mg/dL (11.1 mmol/L) during OGTT or HbA1c ≥6.5% (in the absence of unequivocal hyperglycemia; for these parameters, diagnosis requires two abnormal test results from the same sample or in two separate test samples) or a random plasma glucose ≥200 mg/dL (11.1 mmol/L)), prediabetes state (impaired fasting glucose (fasting plasma glucose concentrations from 100 mg/dL (5.6 mmol/L) to 125 mg/dL (6.9 mmol/L)), or impaired glucose tolerance (2-h plasma glucose levels during OGTT from 140 mg/dL (7.8 mmol/L) to 199 mg/dL (11.0 mmol/L)) or HbA1c from 5.7% to 6.4%) [2], or studies which included participants where the majority had at least two characteristics of metabolic syndrome (waist circumference ≥102 cm for men and ≥88 cm for women, triglyceride levels ≥150 mg/dL (1.7 mmol/L), high-density lipoprotein (HDL) cholesterol <40 mg/dL (1.0 mmol/L) for men and <50 mg/dL (1.3 mmol/L) for women, hypertension or blood pressure ≥130/85 mmHg, or fasting plasma glucose ≥100 mg/dL (5.6 mmol/L)) [24], with no restrictions on age, gender, and race/ethnicity of study participants, location of study, or sample size.

The exclusion criteria were as follows:

- Types of studies: non-RCTs, uncontrolled trials, observational studies (e.g., ecologic study, cohort study, case–control study, case reports, case series, editorials, commentaries, letters to the editor, qualitative research), conference papers, or publications available only in abstract form (no possible contact with authors);
- Population: studies conducted in animal models or studies performed in healthy subjects or a specific group of patients (e.g., pregnant or breastfeeding women).

Any RCTs that assessed the effect of the Paleolithic diet on glucose and insulin homeostasis qualified for consideration.

### 2.3. Quality Assessment

**Figure 1:**
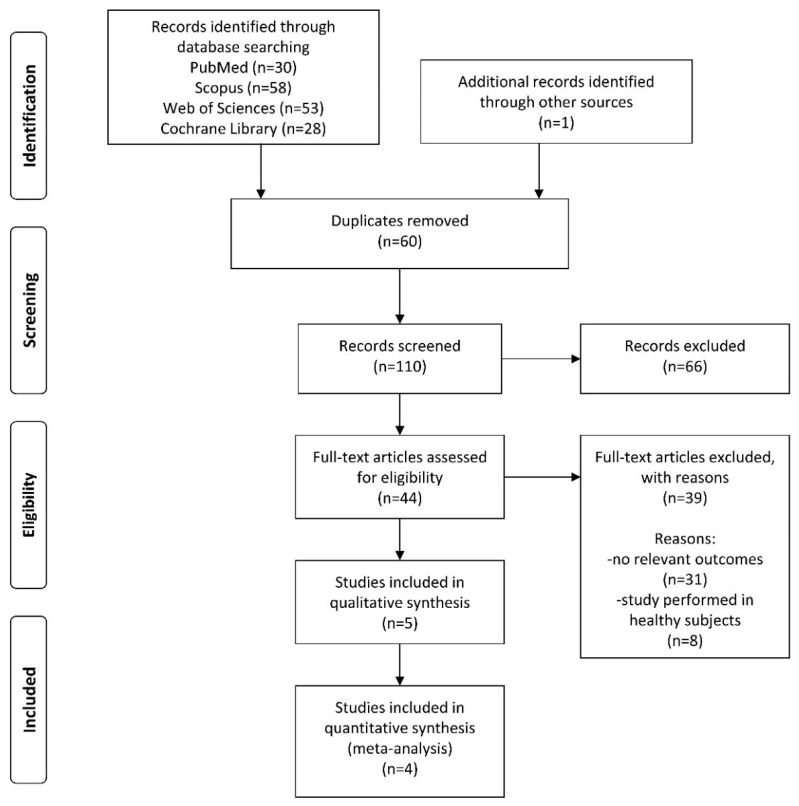
Process of the search.

Three investigators (M.J., A.J., and B.K.) evaluated each article independently in the three main stages of the extraction process (Figure 1). Firstly, article titles were screened, followed by abstracts and finally full texts for eligibility for inclusion in the systematic review and meta-analysis. Disagreements were resolved by discussion between the investigators and included a discussion with two other authors (C.S. and J.W.) until a consensus was reached. All investigators agreed on the final decision of the studies to be included. Primary authors of relevant articles were contacted directly if the data sought were unavailable or if the study was only published in abstract form.

### 2.4. Data Extraction

Eligible studies were reviewed, and the following data were independently extracted by three authors (M.J., A.J., and B.K.):

- General information: first author’s name, publication year, country;
- Study characteristics: study design and method of blinding;
- Characteristics of study participants: sample size (total sample size and number of subjects in each group), age, sex, body mass index (BMI), body weight, ethnicity, and health status (diabetes mellitus type 1, diabetes mellitus type 2, impaired fasting glucose, and impaired glucose tolerance or other);
- Type of intervention: type of diet, the macronutrient composition of diet (the energy value of the diet, percentage energy from carbohydrate, protein and fat, dietary fiber intake (g/day)), recommended and excluded food products, time of intervention, duration of intervention;
- Pre- and post-intervention fasting glucose and insulin levels, HbA1c values, the area under the curve (AUC; 0–120 min) for glucose and insulin during OGTT and homeostasis model assessment of insulin resistance (HOMA-IR).

### 2.5. Risk of Bias

Risk of bias was assessed independently by two authors (M.J. and A.J.) using the Cochrane Collaboration tool, where the following domains are included: selection bias, performance bias, detection bias, attrition bias, reporting bias, and other bias. Criteria for low risk, unclear risk, and high risk of bias per the Cochrane Handbook for Systematic Reviews of Interventions were used [25].

### 2.6. Data Analysis

Subjects were categorized according to the BMI cut-off (BMI = body weight (kg)/body height (m^2^)) values proposed by the World Health Organization for adolescents and adults [26]. According to these criteria, underweight was defined as BMI <18.5 kg/m^2^, normal body weight as 18.5–24.9 kg/m^2^, overweight as 25.0–29.9 kg/m^2^, and obesity as ≥30.0 kg/m^2^.

The ADA recommendations [2] were used to assess fasting glucose and insulin levels, as well as AUC 0–120 for glucose and insulin. Impaired glucose tolerance is defined as plasma concentrations of glucose at 120 min in the OGTT ranging from 7.8 to 11.0 mmol/L, while impaired fasting glucose is defined as fasting glucose levels from 5.6 to 6.9 mmol/L, normal glucose tolerance is defined as glucose levels at 120 min in the OGTT <7.8 mmol/L, and normal fasting glucose is defined as fasting glucose levels ranging from 3.9 to 5.5 mmol/L. Diabetes mellitus was diagnosed when fasting glucose levels were ≥7.0 mmol/L or glucose levels at 120 min in the OGTT were ≥11.1 mmol/L or HbA1c ≥6.5%. A reference range for fasting insulin of <174 pmol/L was assumed [27]. The changes in the HOMA-IR index during the intervention period were used to assess the alterations in insulin resistance within the studied populations. According to ATP III-Met, we defined cut-off values of HOMA-IR for the diagnosis of insulin resistance as ≥1.8 [28].

### 2.7. Outcomes

Our primary outcomes were post-intervention fasting glucose, insulin levels, HbA1c values, and HOMA-IR index. Our prespecified secondary outcomes were AUC 0–120 for glucose and insulin.

### 2.8. Statistical Analysis

Data are presented as means ± standard deviations (SD). We undertook data synthesis, including a calculation of effect sizes with 95% confidence intervals (CIs), using fixed-effects models (if no heterogeneity was present) and random-effects models (to analyze outcomes moderate and high with heterogeneity) with inverse variance weighting [29]. A meta-analysis of the studies was carried out when at least two studies were included that analyzed data for the specific outcome. Standardized mean differences (SMDs) were used as a summary statistic to allow comparison of effect sizes across studies. The SMD is estimated from the difference between the mean outcome values of the intervention and control groups divided by the pooled SD of the outcome values. Forest plots were generated to illustrate the study-specific effect sizes along with 95% CI [25]. Sensitivity analyses were also performed by removing each study one by one and recalculating the pooled estimates. Due to a small number of studies included in this meta-analysis, subgroup analysis was not performed.

Heterogeneity between studies was evaluated using Cochran Q statistics; *p* < 0.1 indicates significant heterogeneity. The I^2^ test was also used to evaluate consistency between studies in which a value <25% indicates a low risk of heterogeneity, 25%–75% indicates a moderate risk of heterogeneity, and >75% indicates a high risk of heterogeneity [30].

All analyses were performed using the Comprehensive Meta-Analysis software, version 3.0 (Biostat, Inc., Englewood, NJ, USA). A *p*-value <0.05 was considered to be statistically significant.

## 3. Results

### 3.1. Search Results

A flow chart of the selection process is shown in Figure 1. The electronic database search identified 110 publications, but screening titles and abstracts excluded 66 articles. The full texts of 44 papers were retrieved, of which 39 papers were excluded from the meta-analysis due to a lack of relevant outcomes or because the study included healthy subjects only. Consequently, four studies were included in this meta-analysis [9,13,16,17,18], one of which had two references related to the same population and the same intervention but reported on different outcomes [9,16].

### 3.2. Characteristics of Included Studies

The characteristics of the included studies are provided in Table 1. These studies were published between 2007 [17] and 2016 [16], and three of them were conducted as RCTs with parallel group designs [13,17,18], whereas one study was designed as a crossover RCT [9,16]. The time of intervention ranged from two weeks [13] to 12 weeks [9,16,17].

In the Paleolithic group, average energy intake varied from 1344 ± 521 kcal [17] to 2079 kcal [13]. Protein provided from 24% [9,13,16] to 28% [17] of energy. In addition, 27% [17] to 41% [13] of energy came from fat. Intake of carbohydrate was in the range from 32% [9,13,16] to 40% [17] of energy. Fiber intake varied from 21.4 ± 13.2 g [17] to 34 g per day [13]. One study did not provide information about total caloric intake and macronutrient composition of the diet [18]. In all included studies, the Paleolithic diet was based on lean meat, fish, fruit, vegetables, eggs, and nuts. The Paleolithic diet generally does not contain dairy products, cereal grains, legumes, refined fats, salt, and sugar. These products were also excluded from the Paleolithic diet in the included studies [9,13,16,17,18].

In the control group, mean energy intake ranged between 1795 ± 306 kcal [17] and 2079 kcal [13], while the average dietary macronutrient composition of the diets was as follows: protein 17% [13] to 20% [9,16,17] of energy, fat 25% [17] to 34% [9,16] of energy, and carbohydrate 42% [9,16] to 52% [17] of energy. Fiber intake ranged from 26 ± 8 g [9,16] to 28 g per day [13]. In one study, the reference diet was based on the guidelines for a healthy diet of the Dutch Health Council [13]. In short, the guidelines recommend intake of more plant-based and less animal-based foods. A reduction of the intake of sugar-based beverages is also emphasized. In this study, the duration of the intervention was two weeks. In another study, the diabetes diet provided evenly distributed meals for 12 weeks with an increased intake of vegetables, root vegetables, wholegrain bread, and other wholegrain cereal products, fruits, and berries. Overall, the emphasis was on increasing dietary fiber, and decreasing intake of total fat with more unsaturated fat being used [9,16]. In another study, the control group received the Mediterranean diet for 12 weeks based on wholegrain cereals, low-fat dairy products, potatoes, legumes, vegetables, fruits, fatty fish, and refined fats rich in monounsaturated fatty acids and alpha-linolenic acid [17], while Masharani et al. [18] provided a diet based on recommendations by the ADA for three weeks. This diet consisted of cereal grains, dairy, or legumes, moderate salt intake, low-fat dairy, whole grains, and legumes.

In two studies, meals were supplied by researchers [13,18]. Boers et al. [13] designed both diets as seven consecutive daily menus (breakfast, lunch, dinner, and snacks). Masharani et al. [18] divided the diets into three meals and three snacks, all prepared by the research center kitchen staff. Participants ate some of the meals in the research center, and the rest of the meals were packed for take-out. In two other studies, participants received written dietary advice and food recipes [9,16,17]. In three studies, to ensure compliance with the intervention, subjects were requested to keep records of the food consumed [9,13,16,17]. In addition, in one study, subjects were encouraged every other day by telephonic contact with their personal coach to complete all meals and to discuss their progress, body weight fluctuation, possible physical and psychological discomforts, or adverse events [13]. In all studies, the emphasis was placed on the prevention of weight loss during the intervention [9,13,16,17,18]. However, at the end of the intervention period, body weight significantly decreased in both groups (−1.5 kg vs. −1.2 kg; −5.0 kg vs. −4.1 kg; −0.9 kg vs. −1.4 kg) for three of the included studies [9,16,17,18]. In addition, two studies observed significant differences between post-intervention body weight, with lower body weight noted in the Paleolithic group [9,13,16].

### 3.3. Characteristics of Study Participants

The characteristics of the study participants are shown in Table 2. In total, 98 subjects were included in the meta-analysis. All studies were conducted in adult populations [9,13,16,17,18]. The average age of study participants ranged from 52.0 ± 10.2 years [13] to 66.0 ± 6.0 years [9,16] in the Paleolithic diet group, and similar values were observed in the control group. Two studies were conducted in Sweden [9,16,17], one in the Netherlands [13], and one in the United States of America (USA) [18]. One study included only men [17], and three studies included both men and women [9,13,16]. In one study, the information about the sex of study participants was not indicated [18]. The mean value of BMI ranged between 28.0 ± 4.0 kg/m^2^ [9,16] and 33.7 ± 5.9 kg/m^2^ [13]. Most of the studies were conducted in Caucasian populations (89% of total study participants) [9,13,16,17,18], but Asian (5% of total study participants) [13,18], African American (3% of total study participants), and Hispanic subjects (5% of total study participants) [18] were also included. Two studies included subjects with type 2 diabetes mellitus [9,16,18], one study recruited subjects with ischemic heart disease plus either glucose intolerance or type 2 diabetes [17], and one study was conducted in subjects with at least two characteristics of the metabolic syndrome (of which 78% of subjects in the Paleolithic group and 44% of subjects in the control group had fasting glucose levels ≥5.6 mmol/L) [13].

### 3.4. The Effect of the Paleolithic Diet on Fasting Glucose Levels

**Figure 2:**
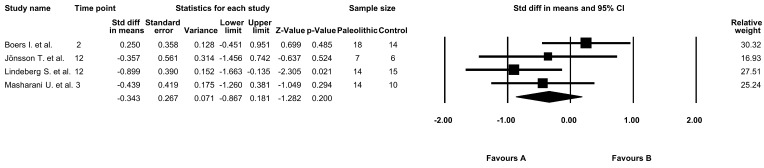
Forest plot comparing fasting glucose levels between the diets (favors A—Paleo group; favors B—control group).

The effect of the Paleolithic diet on glucose levels was analyzed in four studies included in this meta-analysis [9,13,17,18]. The average baseline blood glucose concentrations ranged from 6.1 ± 0.8 mmol/L [13] to 8.4 ± 4.2 mmol/L [18] in the Paleolithic group and were similar to the values observed in the control group. Following the intervention period, the mean glucose concentrations decreased in both groups in all studies [9,13,17,18]. However, the results were statistically significant only in one study for the Paleolithic group [17] (Table 3). On the other hand, three studies noted significant differences between post-intervention fasting glucose levels in the Paleolithic and the control groups [9,17,18]. Nevertheless, this meta-analysis did not report a significant difference between the effect of the Paleolithic diet and the control diet on glucose concentrations (random-effects model, SMD: −0.343, 95% CI: −0.867, 0.181, *p* = 0.200, Figure 2) and indicated a moderate risk of heterogeneity among the included studies (Q-value= 4.828, *p* = 0.185, I^2^ = 37.857%).

### 3.5. The Effect of the Paleolithic Diet on Fasting Insulin Levels

**Figure 3:**
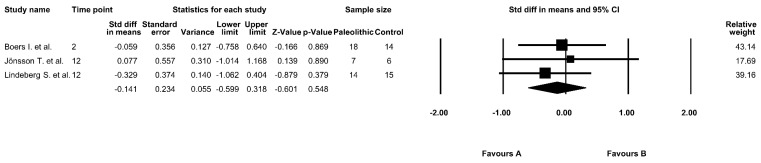
Forest plot comparing fasting insulin levels between the diets (favors A—Paleo group; favors B—control group).

The effect of the Paleolithic diet on insulin concentrations was evaluated in three studies included in this meta-analysis [9,13,16,17]. At baseline, in the Paleolithic group, the mean fasting insulin concentrations ranged from 82.64 ± 38.19 pmol/L [13] to 118.00 ± 53.00 pmol/L [9,16]. After the intervention, the mean fasting insulin levels decreased in both groups in all studies [9,13,16,17]; however, there were significant differences between pre- and post-intervention insulin levels in only two studies in the Paleolithic group [9,16,17] (Table 3). However, the results of this meta-analysis showed no significant differences between the effect of the Paleolithic diet and the control diets on insulin levels (fixed-effects model, SMD: −0.141, 95% CI: −0.599, 0.318, *p* = 0.548, Figure 3). In addition, we observed a very low and insignificant risk of heterogeneity among the included studies (Q-value = 0.459, *p* = 0.795, I^2^ = 0.000%).

### 3.6. The Effect of the Paleolithic Diet on HOMA-IR

**Figure 4:**
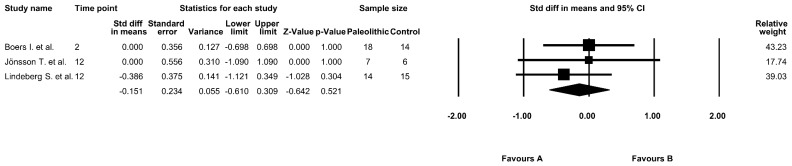
Forest plot comparing homeostasis model assessment of insulin resistance (HOMA-IR) between the diets (favors A—Paleo group; favors B—control group).

The effect of the Paleolithic diet on the HOMA-IR index was analyzed in three selected studies [9,13,17]. At baseline, in two studies, the mean values of the HOMA-IR index were equal or exceeded a value of 1.8, indicating insulin resistance [9,13]. In all studies, the values of the HOMA-IR index decreased after the intervention period [9,13,17]; however, the results were significant in only two studies in the Paleolithic diet group [9,17] and in one study in the control group [9,17] (Table 3). In addition, the results from the meta-analysis did not show any significant differences between the effect of the Paleolithic diet and the control diets on HOMA-IR index (fixed-effects model, SMD: −0.151, 95% CI: −0.610, 0.309, *p* = 0.521, Figure 4), with low to no significant heterogeneity among the included studies (Q-value = 0.645, *p* = 0.724, I^2^ = 0.000%).

### 3.7. The Effect of the Paleolithic Diet on HbA1c Values

**Figure 5:**
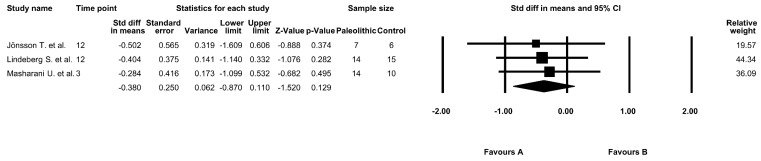
Forest plot comparing glycated hemoglobin (HbA1c) values between the diets (favors A—Paleo group; favors B—control group).

The effect of the Paleolithic diet on HbA1c values was assessed in three studies [9,17,18]. At baseline, mean HbA1c values in the Paleolithic group ranged from 4.76 ± 0.26% [17] to 7.30 ± 2.10% [18]. A significant decrease in the HbA1c values was noted after the intervention period in two studies in both the Paleolithic group and the control group [9,18]. However, the meta-analysis did not confirm significant differences between the effect of the Paleolithic diet and other types of diets on HbA1c values (fixed-effects model, SMD: −0.380, 95% CI: −0.870, 0.110, *p* = 0.129, Figure 5) and indicated a low risk of heterogeneity (Q-value = 0.104, *p* = 0.949, I^2^ = 0.000%).

**Figure 6:**
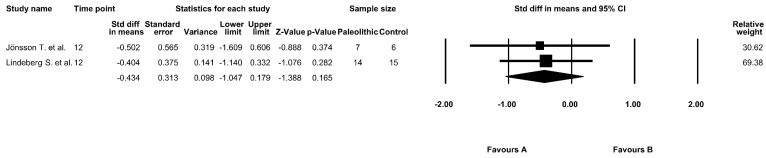
Forest plot comparing HbA1c values between the diets in studies with an intervention period of at least 12 weeks (favors A—Paleo group; favors B—control group).

HbA1c values provide information about average glucose concentrations over the past three months. Therefore, it was suggested that this marker is not reliable in studies with an intervention period shorter than three months [31]. In this meta-analysis, we performed a separate analysis for studies with an intervention period of at least 12 weeks. Nevertheless, the results of this analysis showed no significant differences between the effect of the Paleolithic diet and control diets on HbA1c values (fixed-effects model, SMD: −0.434, 95% CI: −1.047, 0.179, *p* = 0.165, Figure 6) and indicated a low risk of heterogeneity (Q-value = 0.021, *p* = 0.885, I^2^ = 0.000%).

### 3.8. The Effect of the Paleolithic Diet on AUC 0–120 Glucose Levels

**Figure 7:**
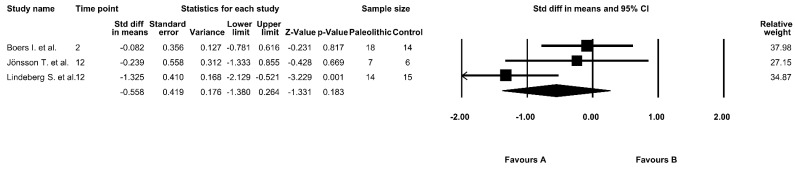
Forest plot comparing area under the curve (AUC) 0–120 glucose levels between the diets (favors A—Paleo group; favors B—control group).

The effect of the Paleolithic diet on AUC 0–120 glucose levels was analyzed in three studies [9,13,17]. At baseline, the mean AUC 0–120 glucose levels in the intervention groups ranged from 263 ± 208 mmol/L × min [13] to 1498 ± 227 mmol/L × min [9]. After the intervention period, the AUC 0–120 glucose levels decreased in all studies [9,13,17]. However, the results were significant only in the Paleolithic diet in two studies [9,17] (Table 4). However, our meta-analysis showed no significant differences between the effect of the Paleolithic diet and the control diets on AUC 0–120 glucose levels (random-effects model, SMD: −0.558; 95% CI: −1.380, 0.264; *p* = 0.183, Figure 7) and indicated a moderate risk of heterogeneity (Q-value = 5.598, *p* = 0.061, I^2^ = 64.271%).

### 3.9. The Effect of the Paleolithic Diet on AUC 0–120 Insulin Levels

**Figure 8:**
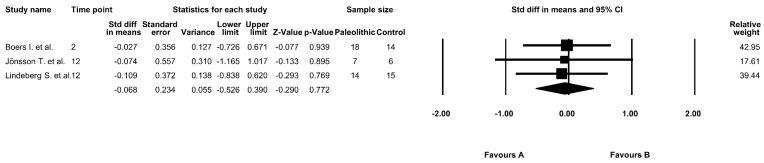
Forest plot comparing AUC 0–120 insulin levels between the diets (favors A—Paleo group; favors B—control group).

Three studies analyzed the effect of the Paleolithic diet on AUC 0–120 insulin levels [9,13,17]. The baseline average AUC 0–120 insulin concentrations in the Paleolithic diet were in the range of 35,000 ± 13,000 pmol/L × min [9] to 80,500 ± 41,100 pmol/L × min [17]. After the intervention period in the Paleolithic group, AUC 0–120 insulin levels decreased in all included studies [9,13,17]. However, the results were significant in only one study both in the Paleolithic group and in the control group [17]. Our meta-analysis showed no differences in the effect between diets on AUC 0–120 insulin levels (fixed-effects model, SMD: −0.068, 95% CI: −0.526, 0.390, *p* = 0.772, Figure 8) and noted a low risk of heterogeneity among the included studies (Q-value = 0.025, *p* = 0.987, I^2^ = 0.000%).

### 3.10. Risk of Bias

Risk of bias is presented in Table 5. Because blinding is impossible in dietary intervention studies, the blinding of subjects and researches was not considered. Most of the included studies were classified as a good quality [9,13,16,17]. However, one study [18] was of fair quality because it did not report methods of allocation concealment and random sequence generation.

## 4. Discussion

The present meta-analysis demonstrates that the Paleolithic diet did not differ from other types of diets commonly perceived as healthy regarding its effect on fasting glucose and insulin concentrations, AUC 0–120 glucose and AUC 0–120 insulin levels, HbA1c values, and the HOMA-IR index.

Despite the fact that, at the end of the intervention period, a decrease in fasting glucose concentrations in the Paleolithic group was observed in most of the studies included in this systematic review [9,13,17,18], our meta-analysis did not show significant differences between the effect of the Paleolithic diet and control diets on fasting glucose levels. Similar results were observed in the previous meta-analysis by Manheimer et al. [8] who also found that the Paleolithic diet did not significantly improve fasting glucose levels. Nevertheless, previous studies showed that a low-carbohydrate diet can contribute to an improvement in fasting glucose levels. In addition, this effect was pronounced in subject with type 2 diabetes [32,33]. Recently, Otten et al. [34] also observed that the Paleolithic diet decreased fasting glucose concentrations in overweight and obese subjects with type 2 diabetes mellitus; however, the authors reported no differences between patients following the Paleolithic diet with standard-care exercise recommendations and the Paleolithic diet together with a 3-h weekly supervised exercise training (−17% vs. −26%). The lack of differences between the effect of the Paleolithic diet and control diets on glucose concentrations observed in this meta-analysis might be partly explained by the negative effect of the Paleolithic diet on microbiota composition. Indeed, it is now well established that an imbalanced gut microbiota is linked to host glycemic control impairment and type 2 diabetes development [35]. In addition, Genoni et al. [36] suggested that long-term adherence to the Paleolithic diet may not be beneficial for gut health, due to the association with lower relative abundances of known beneficial bacterial genera, and the increased relative abundance of trimethylamine-*N*-oxide producing genus *Hungatella*. This is, however, only a speculation since we did not have access to data relating to the gut microbiome.

The previous meta-analysis conducted by Ghaedi et al. [37] reported that the Paleolithic diet could significantly decrease anthropometric parameters, including body weight, waist circumference, BMI, and fat mass. These results were also confirmed by Manheimer et al. [8], who pointed out that Paleolithic nutrition was more effective in reducing body weight in comparison to the control diet. Previous studies also reported that the Paleolithic diet is more satiating than other types of diets. The composition of the diet is likely to be an important factor in satiety and body weight management. It was suggested that the high protein content of a diet might increase satiety and weight loss [13,38]. Body weight is also an important factor that might affect fasting insulin levels. In fact, body weight reduction is reported to significantly decrease fasting insulin concentrations [39]. Here, we observed that the Paleolithic diet did not affect fasting insulin levels when compared to the control group. It is possible that these results could be explained by similar body weight changes after the intervention period observed in both groups. Indeed, in three studies included in this meta-analysis, body weight significantly decreased in both groups after the intervention period [9,16,17,18]. However, two studies observed significant differences between post-intervention body weight, with lower body weight noted in the Paleolithic group [9,13,16].

It is well known that fasting insulin is associated with insulin resistance, which is an essential factor in developing type 2 diabetes. Furthermore, insulin resistance is also implicated in obesity, hypertension, cancer, or autoimmune diseases [40]. Insulin resistance is also associated with excess fat, obesity, or altered lipid profiles [41]. High fasting insulin levels are related to the greater resistance of tissues to insulin, which is reflected through the HOMA-IR index [28,42]. Several studies revealed that a diet pattern concentrated mainly on meat, fish, eggs, vegetables, fruits, berries, and nuts might be effective for improving predictors of insulin resistance such as the HOMA index [14,17,19,20,43]. In contrast, our meta-analysis did not show a significant effect of the Paleolithic diet on the HOMA-IR index. It should be noted that insulin resistance is dependent on fasting glucose and insulin levels [44]. Therefore, the lack of significant differences between the effect of the Paleolithic diet and the control diet on HOMA-IR can easily be explained by the lack of significant changes in fasting glucose and insulin levels [10,14].

HbA1c is a glucose homeostasis parameter which is widely used to assess the metabolic control of diabetic subjects [2]. It is strongly associated with severe diabetic complications [45] and can also be used as a screening tool for subjects with prediabetes [2]. The potential of the Paleolithic diet for a decrease in HbA1c values was observed in three studies included in this systematic review [9,17,18]. In addition, a recent meta-analysis showed that a low-carbohydrate diet, followed by a Mediterranean diet and the Paleolithic diet, was ranked as the most optimal dietary approach for the reduction of HbA1c values [46]. Our meta-analysis, however, did not confirm these results. It is possible that the sample size in studies included in this meta-analysis was too small or the intervention period was too short to detect significant differences between groups.

Our meta-analysis did not show any differences between the effect of the Paleolithic diet and the control diet on AUC 0–120 for glucose and insulin. Similar results were recently obtained by Otten et al. [47] in a study conducted in healthy obese women. Otten et al. [47] observed no difference between the effect of the Paleolithic diet and the control diet on AUC 0–120 for glucose and insulin. However, AUC 0–120 for insulin showed a tendency to decline between baseline and 24 months in both intervention groups.

Several limitations should be listed acknowledged this meta-analysis. Firstly, the number of studies included in this systematic review was relatively small, with a limited number of study participants. Secondly, there were many variations between the studies, including the ethnicity of study participants, age, various metabolic stages, methodology, and duration of the intervention period, as well as a different type of diet used by the control group in selected studies. Moreover, we observed differences in macronutrient composition amongst the included studies. Furthermore, in most included studies, there was no information about the training of the persons who performed the dietary education of the study participants [9,16,17]. In addition, some of the referred studies at baseline showed a significant difference between groups [9]. These factors could have influenced the findings and could partly explain why the meta-analysis data show no significant differences between the Paleolithic diet and the control diet. It is important to also note that most of the studies included in this meta-analysis were performed in Caucasian subjects with elevated BMI values. Therefore, our findings cannot be generalized to other ethnicities such as Asians, in addition to populations with lower BMI values. Moreover, we were unable to assess the long-term effect of the Paleolithic diet on glucose and insulin homeostasis.

On the other hand, the strength of this research is that it includes details on the characteristics of the study and study population, as well as the measures taken to reduce the influence of bias in the included studies. Moreover, this is the first meta-analysis to comprehensively compare the effect of the Paleolithic diet with other types of healthy diets on glucose and insulin homeostasis in subjects with altered glucose metabolism.

## 5. Conclusions

In conclusion, this study provides evidence that the Paleolithic diet did not differ from other types of diets commonly perceived as healthy, in its effect on glucose and insulin homeostasis in subjects with altered glucose metabolism. Further RCTs with long-term follow-ups should be applied for future investigations into the potential benefits of Paleolithic nutrition in the setting of diabetes or metabolic syndrome.

## Figures and Tables

**Table 1:** Nutrient and caloric composition of the dietary intervention.

Study	Year	Type of Study	Duration of Intervention (week)	Groups	Subjects (*n*) ^a^	Description	Energy (kcal)	Protein (%)	Fat (%)	Carbohydrate (%)	Fiber (g)	Meals Supplied by Researchers
Boers et al. [13]	2014	RCT, parallel	2	PD group	18	Based on lean meat, fish, fruit, leafy and cruciferous vegetables, root vegetables, eggs, and nuts. Dairy products, cereal grains, legumes, refined fats, extra salt, and sugar were excluded.	2079	24	41	32	34	Yes
				Control group	14	The reference diet was based on the guidelines for a healthy diet of the Dutch Health Council.	2079	17	29	50	28	
Fontes-Villalba et al. [16] ^b^	2016	RCT, crossover	12	PD group	7	Paleolithic diet based on lean meat, fish, fruits, vegetables, root vegetables, eggs, and nuts.	1581 ± 295 ^c,d^	24 ± 3 ^c,d^	39 ± 5 ^c,d^	32 ± 7 ^c,d^	21 ± 8 ^c,d^	No
				Control group	6	Diabetes diet stated that it aimed to provide evenly distributed meals with an increased intake dietary fiber from vegetables, root vegetables, wholegrain bread, and other wholegrain cereal products, fruits, and berries, and a decreased intake of total fat with more emphasis on unsaturated fat.	1878 ± 379 ^c,d^	20 ± 4 ^c,d^	34 ± 6 ^c,d^	42 ± 7 ^c,d^	26 ± 8 ^c^^,d^	
Jönsson et al. [9] ^b^	2009	RCT, crossover	12	PD group	7	Based on lean meat, fish, fruit, leafy and cruciferous vegetables, root vegetables, eggs, and nuts, while excluding dairy products, cereal grains, beans, refined fats, sugar, sweets, soft drinks, beer, and extra addition of salt.	1581 ± 295 ^c,d^	24 ± 3 ^c,d^	39 ± 5 ^c,d^	32 ± 7 ^c,d^	21 ± 8 ^c,d^	No
				Control group	6	Diabetes diet, meals with increased intake of vegetables, root vegetables, wholegrain bread and other wholegrain cereal products, fruits, and berries, and decreased intake of total fat with more unsaturated fat.	1878 ± 379 ^c,d^	20 ± 4 ^c,d^	34 ± 6 ^c,d^	42 ± 7 ^c,d^	26 ± 8 ^c,d^	
Lindeberg et al. [17]	2007	RCT, parallel	12	PD group	14	Based on lean meat, fish, fruits, leafy and cruciferous vegetables, root vegetables (including restricted potatoes), eggs, and nuts.	1344 ± 521 ^c^	27.9 ± 6.8 ^v^	26.9 ± 6.4 ^c^	40.2 ± 8.3 ^c^	21.4 ± 13.2 ^c^	No
				Control group	15	Consensus (Mediterranean diet) diet based on wholegrain cereals, low-fat dairy products, potatoes, legumes, vegetables, fruits, fatty fish, and refined fats rich in monounsaturated fatty acids and alpha-linolenic acid.	1795 ± 306 ^c^	20.5 ± 3.6 ^c^	24.7 ± 4.3 ^c^	51.7 ± 5.3 ^c^	26.8 ± 7.4 ^c^	
Masharani et al. [18]	2015	RCT, parallel	3	PD group	14	The Paleolithic diet consisted of meat, fish, poultry, eggs, fruit, vegetables, tree nuts, canola oil, mayonnaise, and honey. Excluded dairy products, legumes, cereals, grains, potatoes, and products containing potassium chloride. A series of ramp diets (with increasing levels of potassium and fiber) were developed. Ramp 1 diet was 1 day, ramp 2 for 3 days, and ramp 3 for 3 days.	N/A	N/A	N/A	N/A	N/A	Yes
				Control group	10	Non-Paleolithic-type diet consisting of cereal grains, dairy, or legumes, moderate salt intake, low-fat dairy, whole grains, and legumes; no ramp up for the American Diabetes Association (ADA) diet. Diet based on recommendations by the ADA.						

^a^ Number of participants who completed the study/number of participants who were included in the analysis. ^b^ Studies conducted on the same population. ^c^ Mean and standard deviation. ^d^ Based on Jönsson et al. [9]. RCT—randomized controlled trial; N/A—not available; PD—Paleolithic diet.

**Table 2:** Characteristics of the included studies and the study populations.

Study	Country	Analysed Groups	Age (years) Mean ± SD	Sex (% of Women)	BMI (kg/m^2^) Mean ± SD	Body Weight (kg) Mean ± SD		Race/Ethnicity (%)	Health Status
						Preintervention	Postintervention		
Boers et al. [13]	The Netherlands	PD group	52.0 ± 10.2	72%	33.7 ± 5.9	98.0 ± 18.2 *	95.3 ± 17.5 *	Caucasian 100%	At least two of the following characteristics of metabolic syndrome: waist circumference ≥102 cm for men and ≥88 cm for women, triglycerides ≥1.7 mmol/L, HDL cholesterol <1.0 mmol/L for men and <1.3 mmol/L for women, blood pressure ≥130/85 mmHg or medication, fasting plasma glucose ≥5.6 mmol/L
		Control group	55.4 ± 9.0	75%	29.8 ± 4.9	86.0 ± 14.2	84.3 ± 12.5	Caucasian 87.5%, Asian 12.5%	
Fontes-Villalba et al. [16] ^a^	Sweden	PD group	66.0 ± 6.0	14%	28.0 ± 4.0	92.0 ± 20.0	81.0 ± 13.0 *^,#^	Caucasian 100%	Subjects with type 2 diabetes without insulin treatment
Jönsson et al. [9] ^a^		Control group	63.0 ± 6.0	33%	32.0 ± 8.0	82.0 ± 13.0	84.0 ± 15.0 ^#^		
Lindeberg et al. [17]	Sweden	PD group	65.0 ± 10.0	0%	29.0 ± 4.0	91.7 ± 11.2	88.0 ± 10.7 ^#^	Caucasian 100%	Subjects with ischemic heart disease plus either glucose intolerance or type 2 diabetes
		Control group	57.0 ± 7.0		30.0 ± 2.0	96.1 ± 12.4	93.5 ± 12.8 ^#^		
Masharani et al. [18]	USA	PD group	58.0 ± 8.0	N/A	31.0 ± 5.0	N/A	−2.4 ± 0.7 ^b,#^	Caucasian 62.5%, African American 12.5%, Asian 12.5%, Hispanic 12.5%	Subjects with type 2 diabetes
		Control group	56.0 ± 13.0		34.0 ± 7.0		−2.1± 1.9 ^b,#^		

^a^ Studies conducted on the same population. ^b^ Delta (value at the end of the intervention period minus value at baseline). BMI—body mass index; HDL—high-density lipoprotein; N/A—not available; PD—Paleolithic diet; USA—United States of America. * Significant difference between PD group and control group; ^#^ significant difference between pre-intervention and post-intervention.

**Table 3:** Effect of the Paleolithic diet on fasting glucose and insulin levels, HbA1c, and HOMA-IR (mean ± SD).

Study	Analyzed Groups	Fasting Glucose (mmol/L)		Fasting Insulin (pmol/L)		HOMA-IR		HbA1c (%)	
		Pre-Intervention	Post-Intervention	Pre-Intervention	Post-Intervention	Pre-Intervention	Post-Intervention	Pre-Intervention	Post-Intervention
Boers et al. [13]	PD group	6.1 ± 0.8	5.7 ± 0.8	82.64 ± 38.19	63.89 ± 34.03	3.30 ± 1.70	2.40 ± 1.60	N/A	
	Control group	5.8 ± 0.7	5.5 ± 0.8	70.83 ± 45.14	65.97 ± 36.80	2.70 ± 1.80	2.40 ± 1.30		
Fontes-Villalba et al. [16] ^a^	PD group	N/A		118.00 ± 53.00 *	69.00 ± 30.00 ^#^	N/A		N/A	
	Control group			75.00 ± 12.00	67.00 ± 20.00				
Jönsson et al. [9] ^a^	PD group	7.1 ± 0.7 *	7.0 ± 1.4 *	118.00 ± 53.00 *	69.00 ± 30.00 ^#^	2.40 ± 1.00	1.40 ± 0.60 ^#^	6.20 ± 0.20	5.50 ± 0.70 *^,#^
	Control group	8.6 ± 1.2	7.5 ± 1.4	75.00 ± 12.00	67.00 ± 20.00	1.60 ± 0.30	1.40 ± 0.40	6.90 ± 0.70	5.90 ± 0.90 ^#^
Lindeberg et al. [17]	PD group ^b^	6.8 ± 1.3	5.2 ± 1.1	102.00 ± 36.00	91.00 ± 32.00	0.62 ± 0.38	0.47 ± 0.33	4.76 ± 0.26	4.61 ± 0.25
	PD group ^c^		5.1 ± 1.0 *^,#^		86.00 ± 36.00 ^#^		0.39 ± 0.36 ^#^		4.64 ± 0.22
	Control group ^b^	7.1 ± 1.8	5.8 ± 1.2	123.00 ± 68.00	100.00 ± 45.00	0.75 ± 0.53	0.55 ± 0.42	4.89 ± 0.79	4.84 ± 0.72
	Control group ^c^		6.2 ± 1.4		101.00 ± 53.00		0.55 ± 0.46 ^#^		4.85 ± 0.69
Masharani et al. [18]	PD group	8.4± 4.2	−1.3 ± 1.4 ^d,^*	N/A		N/A		7.30± 2.10	−0.30 ± 0.49 ^d,#^
	Control group	7.7 ± 2.5	0.6 ± 1.8 ^d^					7.00 ± 1.50	−0.18 ± 0.24 ^d,#^

^a^ Studies conducted on the same population. ^b^ Data after six weeks. ^c^ Data after 12 weeks. ^d^ Delta (value at the end of the intervention period minus value at the baseline. * Significant difference between PD group and control group; ^#^ significant difference between pre-intervention and post-intervention. HbA1c—glycated hemoglobin; HOMA-IR—homeostasis model assessment of insulin resistance; N/A—not available; PD—Paleolithic diet.

**Table 4:** Effect of the Paleolithic diet on AUC 0–120 glucose and AUC 0–120 insulin (mean ± SD).

Study	Year	Analyzed Groups	AUC 0–120 Glucose (mmol/L × min)		AUC 0–120 Insulin (pmol/L × min)	
			Pre-Intervention	Post-Intervention	Pre-Intervention	Post-Intervention
Boers et al. [13]	2014	PD group	263 ± 208	245 ± 199	61,047 ± 43,056	47,729 ± 18,694
		Control group	249 ± 162	262 ± 216	43,542 ± 25,132	48,299 ± 23,368
Jönsson et al. [9]	2009	PD group	1498 ± 227	1398 ± 314 ^#^	35,000 ± 13,000	26,000 ± 14,000
		Control group	1734 ± 128	1478 ± 358	24,000 ± 8000	27,000 ± 13,000
Lindeberg et al. [17]	2007	PD group ^a^	1104 ± 118	877 ± 161 ^#^	80,500 ± 41,100	63,100 ± 30,000 ^#^
		PD group ^b^		807 ± 107 *^,#^		56,100 ± 30,100 ^#^
		Control group ^a^	1145 ± 298	1024 ± 339	69,700 ± 44,700	54,100 ± 37,200 ^#^
		Control group ^b^		1065 ± 250		60,400 ± 46,400

^a^ Data after six weeks. ^b^ Data after 12 weeks. * Significant difference between PD group and control group; ^#^ significant difference between pre-intervention and post-intervention. AUC—area under the curve; N/A—not available; PD—Paleolithic diet.

**Table 5:** Risk of bias summary according to the Cochrane risk of bias tool.

Study	Selection Bias		Performance Bias	Detection Bias	Attrition Bias	Reporting Bias	Quality
	Random Sequence Generation	Allocation Concealment	Blinding of Participants and Personnel	Blinding of Outcome Assessment	Incomplete Outcome Data Addressed	Selective Reporting	
Boers et al. [13]	**+**	−	−	+	**+**	+	Good
Fontes-Villalba et al. [16] ^a^	**+**	−	−	+	**+**	+	Good
Jönsson et al. [9] ^a^	**+**	−	−	+	**+**	+	Good
Lindeberg et al. [17]	+	−	−	+	**?**	+	Good
Masharani et al. [18]	?	−	−	−	**+**	+	Fair

^a^ Studies conducted on the same population; + low risk; ? unclear risk; − high risk.

## Supplementary Materials

The following are available online at https://www.mdpi.com/2077-0383/9/2/296/s1.
